# Exploring the subjective experience of rave party participants in Israel who consume psychedelic drugs: a qualitative inquiry

**DOI:** 10.1186/s12954-023-00908-5

**Published:** 2023-12-06

**Authors:** Yula Milshteyn, Moshe Bensimon

**Affiliations:** Department of Criminology, Bar-Ilan University, Ramat Gan, Israel

**Keywords:** Drug policy, Harm reduction, Rave parties, Rave music, Stigma, Interpretative phenomenological analysis, Psychedelic drugs

## Abstract

**Background:**

Rave music parties (RMP) are a world-wide socio-cultural phenomenon, where people listen to rave music while frequently consuming psychedelic drugs. Epidemiological studies have emphasized the hazardous consequences following the consumption of psychedelic drugs at RMP, and qualitative studies have shown social and psycho-spiritual experiences. Yet, phenomenological inquiry into subjective experiences of attendees is scant. This study aimed to examine physical, emotional, perceptual and social experiences of RMP participants in Israel, and their view on Israel’s policy toward rave events. In addition, the study aimed to contribute useful information for policymakers and society on rave music and psychedelic drugs experiences at RMP.

**Method:**

Interpretative phenomenological analysis was used to analyze transcriptions of semi-structured interviews with 27 individuals attending RMP regularly and consume psychedelic drugs.

**Results:**

Analysis revealed four significant themes: the first theme, *the impact of Israel’s drug policy on participants’ sense of safety,* relates to participants’ sense of insecurity and anxiety at Israeli RMP due to government drug ban policy. The second theme, *the stigma on rave culture,* relates to participants’ perception regarding the stigma on rave culture in law enforcement agencies and in society in general. The third theme, *negative experiences,* describes short-term experiences after consuming psychedelics at RMP, including hallucinations and disorientation. The fourth theme, *positive experiences,* describes positive sensory, emotional and self/world attitudinal aspects after consuming psychedelics at RMP. Sensory experiences included intensified auditory, visual and tactile experiences; emotional experiences included positive feelings toward others, reduced stress and ability to vent difficult emotions; self/world attitudinal aspects included self-acceptance, higher appreciation of life and connectedness to nature.

**Conclusions:**

The study highlights RMP participants’ sense of insecurity due to Israel’s strict drug policy and absence of harm reduction strategies at rave scenes. The study also notes participants’ experience of stigmatization as drug addicts by society and law enforcement agencies. Reducing police presence and adopting harm reduction policies at rave scenes in Israel may increase participants’ sense of security, reduce stigmatization and decrease overdose risk. Hence, the findings may contribute to new knowledge useful for policymakers and society concerning RMP and the use of psychedelics.

## Introduction

Rave music parties (RMP) are dance events that take place during the night and may last sometimes up to 12 h or longer [1]. RMP are usually held outdoors, in nature, on weekends, in clandestine places like deserts, forests and deserted beaches [2]. They typically feature DJ (disc jockey) performances of electronic loud and fast (usually 120–150 beats per minute) “rave” music [3]. Secret locations are chosen for being optimally situated far away from law enforcement detection, thus enabling loud music playing and drug consumption [4]. By immersing in nature, rave party attendees enter a transcendental space, where they may experience feelings of awe and shared identity transformation, a collective altered state of consciousness and a strong connection to others, to nature and to the universe as a whole [5]. A distinctive feature of RMP is the wide-spread consumption of classic psychedelic/hallucinogens drugs (“mind-altering”) [6], including psilocybin (O-phosphoryl-4-hydroxy-N, N-dimethyltryptamine), (a.k.a magic mushrooms) and *N*,*N*-dimethyltryptamine (ayahuasca/DMT) and the hallucinogen lysergic acid diethylamide (LSD) [7]. The use of 3,4-Methyl-enedioxy methamphetamine (known as MDMA/ecstasy or “Molly”) is highly prevalent at RMP [8]. Although not considered a classic psychedelic, MDMA is an amphetamine-like compound, also shows hallucinogenic-like effects and can be considered an intermediate substance between a pure stimulant like D-amphetamine and a pure hallucinogenic drug like LSD [9]. The common denominator of these psychoactive compounds is the agonist activity of the serotonin 2A receptor [7].

Rooted in the 1980s, rave music culture has become a world-wide socio-cultural phenomenon, with venues in destinations as diverse as Moscow, Thailand, Spain, South and North America, Europe, Australia, Beirut [10] and Israel [11–13]. One of the main components of a rave is its distinctive ethos called ‘PLUR’, an acronym for peace, love, unity and respect [14]. This philosophy is a hallmark of rave party attendees’ collective identity and refers to the shared feelings of solidarity, love, acceptance and respect for the self and each other. RMP are commonly organized in a spirit of inclusiveness, where aggression, judgementalism, racism, sexism and homophobia are frown upon [15].

A large body of quantitative, epidemiological and pharmacological studies using cross-sectional and survey-based research methods have emphasized the magnitude and the psychological, behavioural and medical adverse effects of drugs at RMP [16–18]. Conversely, qualitative anthropological [19], ethnographic [20] and socio-cultural [21] studies have emphasized social and psycho-spiritual experiences showing that RMP facilitated a wide range of psycho-social and cultural processes, such as solidarity and connectedness [22]. Studies described the experience as a collective state of consciousness, an implicit religion [4, 23] and a modern-day ritual [24]. RMP has also been described as an extraordinary, transitory euphoria, a joyful and empathic experience, a festive, carnivalesque event and a “state of flow” [25]. Few recent studies showed that drug-related experiences could be related to participants’ perception of harm reduction approaches and health-care services at RMP [23, 26].

Within the Israeli context, only a handful of ethnographic and sociological studies have been conducted on RMP. Those studies relied mainly on prolonged fieldwork and a participatory-observational methodology [11, 13, 27–29]. Scholars have interpreted the Israeli rave music culture in terms of a collective national identity formation, and a counter-reaction to the pressure caused by the Israeli-Palestinian conflict and by the order imposed by the mandatory military service [11, 27]. Yet, in-depth research regarding the subjective experience of rave party attendees and the meaning they derive from their experiences remains sparse. Thus, the present study strives to expand upon previous research and examine retrospectively, through a phenomenological research paradigm, the subjective experience of individuals participating at RMP in Israel.

### Rave scene in Israel: a brief overview

Israel is one of the countries where rave parties, or trance parties, as they are called in Israel, have attained a high degree of participation and spread [30]. Within the Israeli dance music scene, psychedelic rave music and its sub-genres such as “Full-On” psytrance, are the most played genres and are considered a sub-culture on its own [31]. While Israelis are involved with many styles of electronic dance music (EDM) (e.g., “House” and “Ambient”), their specific contribution to the “Full-On” psytrance (psychedelic trance) genre is immense [11, 28]. Full-On trance is melodic, energetic, with crisp bass-lines and fast tempo (usually 140–148 beats per minute). It is played worldwide by many Israeli rave music artists, like Skazi (Asher Swissa), the duo Astral Projection, Infected Mushroom and DJ Astrix (Avi Shmailov) [11].

Rave music culture was introduced in Israel in the early 1990s by Israeli backpackers who returned from their post-army trips abroad (mostly to the Far East) and wanted to locally re-create what they had experienced abroad [29]. The Israeli rave culture has shifted from being an avant-garde subculture to a widespread, although not entirely legitimate, leisure activity [12]. Rave parties in Israel can get official permits, but participants are not allowed the use of drugs in those parties. An average of 20 raves with police approval are organized in Israel every month, alongside dozens more clandestine raves that are held without a permit [32]. Israeli rave party attendees are primarily secular Jewish youth, with diverse attitudes toward tradition and institutionalized rituals. Overall, Israeli outdoor rave music events, both authorized mass festivals and illegal parties, are estimated to weekly attract as many as 100,000 participants of varied ages and genders, and of diverse social and economic backgrounds [12]. The government and law enforcement agencies play a pivotal role in creating moral panic regarding the Israeli rave scene, as party attendees report repeated aggressive policing at such parties (i.e., brutal enforcement and arrests [27]).

### The effects of psychedelic drugs at RMP

Psychedelic drugs are powerful psychoactive substances that are known for altering perception and mood and affecting numerous cognitive processes [7]. These psychoactive substances induce altered states of consciousness, including the sense of self [33]. The use of psychedelic drugs at RMP was found to cause various short- and long-term adverse outcomes such as nausea, headaches, chest pain, depression, dehydration, dizziness, hyperactivity, life-threatening poisoning, hyperthermia, serotonergic toxicity, overdose and even death [17, 34–39].

Sociological and ethnographic qualitative studies that were mostly conducted in Western countries, have shown that RMP also create positive transpersonal and transformative experiences, such as enhanced positive attitude toward life, self-acceptance and self-love, connectedness to self, world and others [40] and spiritual experiences such as self-healing through an altered state of consciousness, and mystical-like God encounter experiences [23, 41]. Additionally, anthropological studies have found a parallel between RMP and shamanic rituals, conceptualizing the parties as a collectivist ritual dance [42, 43]. Within this context, RMP were found to contribute to participants’ psychological and emotional well-being and enhanced mood [44, 45], while serving as a sacred liminal space that facilitated psycho-spiritual healing processes [46]. In addition, participants reported that the combination of dance and drugs at RMP intensified their awareness of being “a kind of bodily mysticism in which mystical union is experienced on physical, somatic and kinesthetic levels” [p. 78].^41^

### The current study

The above literature review of qualitative ethnographic and socio-cultural research regarding RMP indicates that participation at such parties fostered positive psycho-social [44], transcendental [46] and spiritual experiences [24]. A small body of research on the rave scene in Israel adopted a naturalistic, ethnographic and sociological world view. Thus, studies described the Israeli scene mainly in terms of a collective national and socio-cultural identity formation [13, 28, 29, 47], and in terms of tribalism and a modern day-ritual [11]. Yet, not much research has been done to gain an in-depth understanding of the “lived experience” [48] of Israeli RMP attendees, both in Israel and abroad. Thus, the present study strives to expand upon previous research by employing a phenomenological research paradigm. It explores the subjective experience of Israeli RMP attendees in order to obtain the most authentic meaning of RMP and examine the role of psychedelic drugs as perceived by participants. The research questions addressed in this paper are: What is the physical, emotional, perceptual and social experience of Israeli RMP attendees, and what is their attitude toward Israel’s policy regarding RMP?

## Method

### Research approach

Interpretative phenomenological analysis (IPA) was used in this study. As a qualitative research approach, IPA is well suited for understanding how people make sense of their experiences [49]. It is influenced by symbolic interactionism with its concern for how meanings are constructed by individuals within both their social and personal world [48]. IPA draws upon the fundamental principles of phenomenology and hermeneutics [50]. As such, IPA allows to look closely at how individuals make sense of life experiences (phenomenology) and provides detailed interpretation of the account to understand those experiences. The IPA approach was selected for the current study in an attempt to obtain the most authentic meaning of the RMP experience and the role of psychedelic drugs as it is perceived by party attendees.

### Participants

Twenty-seven Israeli Jewish, RMP attendees, aged 20–50 (*M* = 38.3) participated in this study (nine females and eighteen males). Though rave music scene has been considered a sub-culture of younger ages (15–25) [51, 52], few studies showed that the RMP in Israel attract large numbers of relatively mature and over the age of 30 individuals [11, 26]. Twenty participants had high-school diploma and seven had higher education, of whom six had a bachelor’s degree and one had a doctoral degree. The higher education prevalence of this sample is consistent with other Israeli rave studies [13, 26]. Participants came from different professional backgrounds (nine musicians/music artists, three social event promoters/organizers, two therapists, two advertisers, two housewives and one of each of the following professions: engineer, construction worker, high-tech employee, truck driver, film producer, psychology student, film student, psychologist, public relations promoter). Twenty participants were either married or in a relationship, 12 of which had children. Seven participants were single. Participants’ history of attendance at RMP ranged from 1 to 30 years (an average of 10 parties a year). Six participants reported attending RMP in Europe as well (i.e., Brazil, Portugal, Netherlands and Australia). Inclusion criteria were a minimum age of 18 (for ethical reasons) and a history of attending at least one party a month for at least one year. Fifteen participants consumed LSD at RMP, 10 consumed MDMA and two reported mixing LSD or MDMA with other substances, such as marijuana or alcohol. None of the participants had a history of psychiatric hospitalization.

### Procedure

Purposive criterion sampling [53] was used to identify individuals who have experienced RMP firsthand and could provide detailed information regarding the phenomenon. Accordingly, participant recruitment was conducted through social media channels such as Facebook sites relating to rave parties in Israel. In addition, snowball sampling [54] was used via interviewee referrals. The criterion used for ending the sampling process was categorical saturation, when no new categories or sub-categories emerge in the content analysis [55].

Interviews lasted between 50 and 150 min, with an average of 90 min. All participants agreed to be audio recorded and were guaranteed anonymity. Two interviews were conducted in English, per request of the participants. All other interviews were conducted in Hebrew. All interviews were transcribed verbatim. Due to the Covid-19 pandemic, the interviews were conducted on the Zoom platform. Recent studies suggest that a Zoom platform could be an effective tool for building and maintaining rapport during interviews [56, 57]. It was found to help participants feel at ease, freely express themselves and improve their inter-personal interaction during an interview [58]. The current research was approved by Bar-Ilan University’s Institutional Review Board (IRB). Participants signed informed consent forms prior to each interview. Information about the study and its aim was provided, promising anonymity and the right to withdraw at any stage without consequences.

### Data collection

Data were obtained via semi-structured interviews [59]. An interview guide [60] included open questions covering a variety of issues. Key questions were: how would you describe RMP? What physical experiences do you feel in your body during RMP? What are your thoughts and feelings when attending RMP? If you have used psychedelic drugs at an RMP, how would you describe your experience? How do you think society perceives the rave scene? If you have encountered enforcement agents at an RMP, please describe the experience. The quotes in the findings section were translated from Hebrew to English by a professional linguist.

### Data analysis

The following seven IPA steps for data analysis [48] were used in this study: reading and re-reading the transcribed interviews to immerse in the original data; writing initial notes of free associations and semantic content in the margins; analyzing and sorting the notes according to emergent themes; integrating themes by searching for connections across the emergent themes; bracketing previous themes and keeping an open-mind regarding new data that can be gleaned from each new case; searching for patterns across cases and noting idiosyncratic cases; and using other theories to interpret the data at deeper levels.

### Trustworthiness of the study

Lincoln and Guba [61] defined *trustworthiness* as a way for researchers to have confidence in their findings and to be able to convince readers of the worthiness of their findings. The following strategies were undertaken to minimize possible falsifications and maintain high standards of credibility:

*Reflexivity.* Researchers’ reflective commentary is a strategy to ascertain confirmability of the study and maintain rigor. Accordingly, the researchers kept reflective notes during the interviews and during the data analysis phases. An example of the first author’s (who conducted the interviews) reflexive process is quoted below:

*Music has always played an integral part in my life. However, I did not have a relationship with RMP and psychedelic drugs. I decided to take upon this research project because of my passion to music and curiosity to gain comprehensive knowledge on the experience of the use of psychedelic drugs within the context of RMP. My lack of personal experience with RMP and psychedelics may have affected the process of data collection and analysis. On the one hand, I approached the study with little knowledge on psychedelic drug experience. That may have had an effect on the questions I asked during the interviews. In order to reduce the effect of this drawback, I read many academic materials on the subject, and watched documentaries. On the other hand, as an outsider I probably was less biased by personal perceptions on the subject. It is important to note that I tried to approach the subject with an open mind and a non-judgemental attitude. Conversations with my PhD supervisor (the second author) supported this approach.*

*Peer debriefing.* This is a process in which the collected data are presented to peers for critical review [62]. Accordingly, the interview transcriptions were sent to the second author and to another PhD researcher specializing in qualitative research methods, both of whom reviewed the transcripts and provided feedback regarding the interpretation of the interviews.

*Member checking.* Member checking is the process whereby the interview transcript is returned to participants, asking them to check the transcript for the purpose of enhancing accuracy of data [61]. Accordingly, the initial findings were shared with the interviewees to get their feedback regarding the adequacy of the analysis. Their corrections and comments were then integrated into the final manuscript.

## Results

As can be seen from Table 1, analysis of the interviews revealed four significant themes regarding the subjective experience of Israeli RMP attendees. The first related to *the impact of Israel’s drug policy on participants’ sense of safety* as opposed to how they feel at similar parties in Europe. Participants noted that from their perspective, decriminalization or legalization of drug consumption in European countries, such as Portugal and the Netherlands, has led policymakers to employ harm reduction strategies at RMP, thus creating a sense of safety, both by having police maintaining order and by having health professionals overseeing participants’ well-being. Israel, on the other hand, bans drug use at RMP and its policy is to impose punitive measures on rave party organizers, thus creating a sense of insecurity among participants for fear of unexpected police crackdowns. In addition, the illegal status of drug use in Israel hinders initiatives to employ official harm reduction strategies at RMP. The second theme—*the stigma on rave culture*—relates to how participants perceive the rave culture stigma in society in general and in law enforcement in particular. The third theme—*negative experiences*—describes experiences such as hallucinations and disorientation, following psychedelic consumption at RMP. The fourth theme—*positive experiences*—refers to positive sensory, emotional and attitudinal aspects following psychedelic consumption at RMP. Positive sensory experiences included auditory, visual and tactile experiences; positive emotional experiences included positive feelings toward others, stress reduction, the ability to vent difficult emotions, a time-out from daily stress and relief from social constraints; positive attitudinal aspects included self-acceptance, greater appreciation of life and connectedness to nature.

**Table 1:** Themes regarding the subjective experience of Israeli RMP attendees

Themes and sub-themes	
1. The impact of Israel’s drug policy on participants’ sense of safety 2. The stigma on rave culture 2.1 Social stigma 2.2 Stigma in law enforcement agencies 3. Negative experiences 4. Positive experiences 4.1 The sensory aspect 4.2 The emotional aspect 4.2.1 Positive feelings toward others 4.2.2 Stress relief 4.2.2.1 Venting difficult feelings 4.2.2.2 A time-out from everyday stress 4.2.2.3 Relief from social constraints 4.3 The attitudinal aspects 4.3.1 Self-acceptance 4.3.2 Appreciation of life 4.3.3 Connectedness to nature	

1. The impact of Israel’s drug policy on participants’ sense of safety

Participants who had attended RMP in Europe reported that drug use in Europe is legal and that health care and harm reduction services are always present at the festivals. Participants explained that harm reduction policy in Europe included drug checking stations and educational awareness campaigns, with flyers containing relevant information on drug content and potential consequences. Arik, a 38-year-old hi-tech worker, reported that in his view, if Israel adopted the European drug policy, drug-related death cases at RMP in Israel could be prevented:

*I was at a rave festival in the Netherlands, and I saw drug checking stations that performed chemical analysis of drug purity and compounds. In Israel, the attitude toward drugs is negative because they are illegal. It makes me feel insecure, and I do not feel free to enjoy the rave experience. If Israel adopted the European drug policy and provided stations for medical services and drug analysis at rave parties, drug-related death incidents could be prevented.*

Ella, a 50-year-old musician, mentioned that when she participated at a rave festival in Portugal, she noted that public health services monitored drug use. In addition, services distributed flyers that contained information on how to use drugs, their content (i.e., degree of purity) and which drugs may be mixed together:

*In Portugal, drugs are legalized.*^1^* At the “Boom” festival, there are drug-checking stations for drug quality and purity. Flyers are distributed with information on drug effects, the compounds they contain, and which drugs may be mixed together. If people take drugs at these events, at least they are aware of the potential risks and have the means to check the purity of the drugs. In Israel, at the ‘Neverland’ rave festival, I saw undercover police officers who were searching for drugs. They were scary. It made me feel suspicious and unsafe. Every time I attended a rave party in the past 25 years, I was anxious about the situation where the police would come and arrest me. […] In Portugal, at a rave party of ten thousand attendees, police officers did not search for drugs. Their job was to maintain order.*

Ron, a 50-year-old rave party organizer, reported that when he attended rave music festivals in the Netherlands, law enforcement agencies monitored drug use. He said that a drug awareness educational campaign in Israel could raise awareness among young party attendees who consume illicit drugs, and may reduce consumption of recreational drugs:

*In the Netherlands, at rave parties, there is an educational drug awareness campaign, in which information is distributed regarding drug types, their effect, the correct dosage and adverse consequences of drug abuse. If Israel adopted the European drug policy, it would raise awareness among young rave party attendees, and might reduce peer pressure to consume drugs. […] The police attitude in Israel has made me feel uncomfortable and anxious. I often look behind me, fearfully checking for police who might be there to shut down the party.*

2. The stigma on rave culture

*2.1 Social stigma*

Rafael, a 35-year-old music artist, explained that society mistakenly associates rave culture with drugs as a default. Rave festival attendees are perceived as drug addicts, thus stigmatizing the entire musical scene:

*Society has a stigma on rave culture. When people hear about someone who took drugs at a rave party and died, they immediately stigmatize the whole scene. In truth, at the end of a party, the dance floor is clean as it was at the beginning, but when people read in the news that someone at a rave party left garbage in the area they say: ‘Those drug addicts, they destroy nature’.*

Similarly, Sharon, a 37-year-old advertiser, explained that people misperceive RMP as associated with drugs:

*Society has a misperception about rave culture. Let’s say that popular opinion is not in our favor. People automatically think that rave parties mean drugs, that nature parties mean drug parties. It’s true that drugs exist, but there are also drugs at night clubs and other scenes. Nature parties are called ‘nature parties’ because they are held outdoors. They should not be automatically associated with drugs.*

*2.2 Stigma in law enforcement agencies*

Participants described persecution of rave culture by the police. Arik, who was mentioned above, described how at one rave party, the police arrived and without warning, stopped the music, banned the party, confiscated equipment, wrote down the organizers’ information (ID number and driver’s license) and sent all ravers home:

*I attended a rave party, and the DJ was playing the last music track. Suddenly the police arrived, and without any warning, stopped the music and banned the party in the middle of the track. I was shocked. The police confiscated equipment, the organizer’s phone and documents and sent everyone home.*

Ella, who was mentioned above, described how she attended a small rave party in nature**,** in a settlement of a small community, and the police arrived and arrested the DJ, just because the attendees listened to rave music in nature. Everyone was shocked:

*I attended a small rave party in a Kibutz. We were all dancing and having fun. Suddenly the police arrived. The police officers had frown faces. They arrested the DJ. We were shocked. I have not seen police raids on parties held in night clubs, but a small nature party they do just because we listen to rave music. It is absolutely absurd. The rave culture has been persecuted for almost 30 years.*

3. Negative experiences

Leon, a 27-year-old film student, described how overdosing on LSD at a rave party induced experiences of seeing his ego and hearing voices. He was terrified of losing control:

*I walked toward the wooden stairs and went up the first step. Then everything turned dark, and fear took on a physical form. […] I suddenly heard my ego’s voice. I noticed its facial expressions and its tone. There was my ego. It had a face. It had a voice. I saw it in front of me.*

Tod, a 40-year-old social event promoter, described an episode in which taking LSD at a rave party induced a hallucination of becoming a snowball of light and darkness, good and evil. He was afraid of losing control:

*I felt the music speaking to me. I slowly closed my eyes, but felt that something was not working. I felt the energy growing and that I was losing control. I felt I was turning into a snowball and spinning around. The snowball got bigger and bigger till it turned into a huge snowball of light and darkness, good and evil. It scared me to death. I felt I was losing control.*

Joel, a 28-year-old psychology student, described an episode of auditory hallucination and disorientation after taking LSD at a party:

*The drugs affected me badly. Even now, talking about it, makes me shiver. I had hallucinations after the party. I was at my sister’s house, and I suddenly heard a noise in my ear that sounded like – “shhhh.” I realized those were sounds from the rave music I had heard at the party. I panicked and told my sister I had to leave. I didn’t want her to know I had taken LSD at the party. I got into the car and felt lost. I was in my own city, but I had to type in my girlfriend’s location on Waze to know how to get there. It was weird. I drove, looked left and saw a road junction that I didn’t remember ever seeing before. Eventually, I arrived where I was supposed to meet my girlfriend but the place seemed so foreign, like I was in Paris.*

4. Positive experiences

*4.1 The sensory aspect*

Participants reported that the combination of rave music and psychedelics enabled them to experience positive auditory, visual and tactile experiences. Debbie, a 42-year-old marketing advertiser, described how consumption of MDMA enabled her to experience music in an intensive auditory manner:

*I need substances to connect to the music, and then I hear the music in a different way. I remember being there and thinking – this is how music is supposed to sound like. […] With MDMA, I could feel the rise and fall of the melody on a physical level. I could feel it all over my body. It opened my heart, my ears and my brain and I had goosebumps the whole time. I love going to rave parties because I don’t just hear the music, I feel it with my body. I think it’s part of the intense experience that you can get only when you take psychedelics at a rave party.*

Aaron, a 45-year-old therapist, described the deep visual connection that LSD adds to the musical experience:

*When I take LSD at rave parties and stand next to the speakers, I literally see the music. I visualize it and let the music and the words to go through me. I make love to the music. It is a very powerful experience*

Denis, a 48-year-old music artist, described a synergy between rave music and LSD. He could see the trees dancing, the birds tweeting a melody and feel the grass moving under his feet:

*There is a beautiful synergy between LSD and rave music. The repetitive sounds of rave affect me on the physiological and visual levels. I feel strong vibrations in my inner organs, in my guts. The music enters my veins. I see trees dancing, hear the birds tweeting a melody, and feel the grass moving under my feet.*

Sharon, who was mentioned above, explained how after consuming MDMA at a rave party, she felt the grass moving toward her and touching her and felt the music coming out of the speakers and talking to her:

*When I took MDMA at a rave party, I felt my body moving and saw the grass moving in my direction. I could feel it touching me. I looked at the speakers and the music was talking to me.*

*4.2 The emotional aspect*

*4.2.1 Positive feelings toward the other*. Participants reported that the combination of rave music and psychedelics intensified their positive emotions. Shelly, a 35-year-old housewife, described intensification of positive emotions toward others:

*For me, rave combined with MDMA enhances the whole experience. It intensifies my emotions and penetrates every part of my body. I feel more outgoing, empathic and loving toward others. I hug everyone.*

Beth, a 43-year-old musician, explained that daily exposure to the news makes her perceive others negatively. Participating in rave parties helps change that perception and enables her to see the good in people:

*I consume news so often that it drives me crazy. It changes me from the inside. Rave parties balance my suffering mind. I regain trust in humanity and realize that not all people are bad. I can suddenly perceive people as kind and caring.*

*4.2.2 Stress relief*

*4.2.2.1 Venting difficult feelings.* Participants reported that they were able to vent difficult feelings at RMP, under the influence of LSD. David, a 50-year-old music producer, reported that for him, rave parties created a therapeutic space where he could channel negative feelings and cope with his brother’s death:

*I love dancing in the forest, so for me it is a therapeutic space. The combination of drugs, music and nature frees me from negative energy and channels my emotions through my body. It helped me cope with my brother’s death.*

Rob, a 42-year-old film producer, explained that under the influence of LSD at a rave party, he was able to let go of emotional and mental residues and process a painful event from his past:

*Rave is medicinal for me. I had been burdened with a highly emotional memory from the past. And then, at one rave party, under the influence of LSD, I was finally able to process that memory and free myself from any residual emotions. […] I stood on the dance-floor, in front of the speakers, absorbed the music and channeled my emotions through dance.*

*4.2.2.2. A time-out from everyday stress.* Jeffrey, a 28-year-old public relations worker, reported that his life was under constant stress and anxiety. RMP helped him find relief from daily stress:

*Life is full of stress and anxiety. Every time I watch the news, I feel more anxious – like a pressure cooker. At rave parties, I can loosen up without being constantly afraid of my life exploding in my face.*

Ezra, a 35-year-old truck driver, reported that RMP provided an opportunity for him to disconnect from the stress of daily life:

*Attending RMP and consuming LSD enable me to escape from daily routine, from stress at home and work. For me, raving is a camping-like experience, an opportunity to forget about deadlines and family responsibilities. It is a fun and unlimited enjoyable experience.*

*4.2.2.3 Relief from social constraints.* Participants described the rave scene as a non-judgemental space, where they could experience a self-liberating process from social constraints and social definitions, as depicted by Ron:

*Rave is an expression of true freedom from social constraints and social definitions. It removes all layers of unnecessary energy. I was standing one morning in the middle of a dance-floor, after a long night of dancing, and I was weeping like a baby. Those were tears of joy and delight. I was celebrating my self-liberation. It was an earth-shaking experience.*

Shelly, who was mentioned above, stated that RMP symbolized for her freedom from social constraints and stigmas:

*When I speak of rave party, I mean freedom from social boundaries and definitions. Rave party is an opportunity of self-expression and liberation. I could dance freely and dress up without being judged. Raving means disconnection from social constraints and stigmas.*

*4.3 The attitudinal aspect*

*4.3.1 Self-acceptance*

Participants reported that RMP fostered self-acceptance. Leon, who was mentioned above, explained that he used to criticize and judge himself for years. Attending rave parties enabled him to accept and love himself:

*Rave parties enabled me for the first time in my life to see the beauty in myself, and it was fascinating. I used to judge myself for so many years, and the change was incredibly liberating. I was sucked into this self-acceptance and it grew inside me. I suddenly loved myself for real, for the first time in my life. I ran to the woods, saw a huge tree and hugged it tight. I raised my head up toward the sky and shouted: “I love myself!” And I cried in joy.*

Ruth, a 30-year-old musician, described how attending rave parties enabled her to connect to herself and understand who she really was:

*Rave increased my self-acceptance. Rave parties for me are a way to connect to myself. Before attending rave parties, I hadn’t known the real me. I hadn’t really seen or understood myself. I only realized who I really was when I connected to my true inner self. I learned to let go and to be less judgemental toward myself.*

*4.3.2 Appreciation of life*. Robert, a 40-year-old construction worker, said that taking LSD at RMP helped him appreciate his life, and especially what his family meant to him:

*At rave parties, in nature, under the influence of LSD, when I listen to rave music and look down at the ground, at my feet and the sun – I realize how blessed I am. I have a child to hug, a wife to love. Rave is the light. It has awoken in me the desire to live, to feel and to love my family.*

Ruth, who was mentioned above, described how rave parties heightened her appreciation of life:

*Taking LSD at rave parties gives me the strength to get on with my life. It helps me get up in the morning and appreciate life. I can now understand that the smallest nuances of life are a gift. I realize I live on borrowed time here on Earth. It is difficult for me to hear about people who give up on themselves and decide to end their lives.*

*4.3.3 Connectedness to nature*. Participants described how open-air rave parties, under the influence of psychedelics, made them feel connected to nature. They could disconnect from technology and social media such as Facebook, and turn to nature, listening and connecting to it. Shelly, who was mentioned above, described how dancing in nature, under the influence of drugs, led her to explore the nature surrounding the location of the party and immerse in it:

*Nature opened my heart. When I danced at an open-air rave party under the influence of LSD, I felt free. I felt like a child exploring the world and it triggered all my senses. It was like going on a field trip, immersing in nature, exploring every inch, turning over every rock and stone and discovering the world.*

Robert, who was mentioned above, described how at RMP in a desert, under the effects of psychedelics and combined with rave music, he felt united with the universe which enabled him a meaningful experience:

*Nature means real freedom. At a rave party, in the desert, I danced barefoot. Under the influence of LSD and listening to psychedelic rave music, I felt the sun, the sand touching my skin. The beautiful landscapes and trees made me feel united with the universe and it enabled an amazing meaningful experience.*

Similarly, Arik, who was mentioned above, described how he felt united with the universe in the forest at RMP:

*At rave parties, in the forest, under the influence of LSD and rave music, I felt united with the universe. I felt connected to earth and trees and to the beautiful landscapes. I took a long walk exploring the surroundings. It was an amazing experience.*

## Discussion

The aim of this study was to examine the experience of Israeli RMP attendees regarding the physical, emotional, perceptual and social aspects of their experience, and their view on Israel’s policy toward rave events. Findings emerging from the thematic analysis yielded four main themes. The first refers to *the impact of Israel’s drug policy on participants’ sense of safety.* A country’s drug policy refers to the overall approach taken by its government regarding illicit drug issues, including monitoring drug sales and consumption, and creating regulatory processes to maximize harm reduction [64]. The leading formal drug policy in Israel is the traditional approach of abstinence, probation and punitive measures, based on three main principles: enforcement, treatment and rehabilitation and prevention. Since 1970’s, Israel has implemented several harm reduction intervention and treatment programmes for various groups of drug addicts. These programmes include the methadone maintenance treatment, buprenorphine maintenance treatment, needle and syringe exchange programme services for HIV (human immunodeficiency virus) and special health services for individuals who inject drugs on the street. Yet, Israel still lacks a comprehensive harm reduction policy within the context of RMP [65]. Israel has no official harm reduction at RMP, and this is conspicuous compared to other countries like Canada [66], UK [67], Italy [68], Portugal [69, 70], USA [17, 71] and Australia [72]. Studies show that drug-checking points (chemical analysis of drug compounds) at RMP and health management services may reduce consumption of “non-pure” substances among rave party attendees, thus increasing their sense of security. The feelings of insecurity found among participants in the current study, can be supported by a recent quantitative cross-sectional survey-based study conducted in Israel, which shows that rave party attendees exhibit negative attitudes toward policing at outdoor raves and are reluctant to seek help from the police when in physical or emotional distress [73]. A harm reduction policy may decrease such negative feelings toward policing at outdoor raves.

The second theme refers to the social aspect—*the stigma on rave culture*. It relates to the way participants perceived the stigmatization of rave parties as drug culture by law enforcement agencies and the general public. Stigma is an attribute, behaviour, or reputation that places individuals outside societal norms. It includes alienation and discreditation of such individuals, which results in branding them as having a “spoiled identity” and being less desirable [74]. The current findings add to the literature on stigmatization of people who use drugs by showing that a music subculture can be stigmatized and viewed as dangerous not solely due to the aspect of drug use in that culture, but because of the setting in which drug use occurs. This is similar to stigmatization of other cultures such as street culture, in which homeless people are stigmatized as criminals, drug addicts, deviants and dangerous [75, 76]. The findings concerning stigma on rave culture may be explained through what Stanley Cohen [77] termed *moral panic*, whereby society views subcultures as a social problem. Accordingly, moral panic occurs when a “condition, episode, person or group of persons emerges to become defined as a threat to societal values and interests” (p. 1). Cohen claims that mass media amplifies deviant behaviour, consequently manufacturing stigma. Moral panic often begins with an initial escalation of media attention to an issue that they regard as a deviance from the norm, thus initiating a process of “sensitizing” the public. When the media is interested in influencing public opinion regarding a certain issue, it tends to exaggerate relevant details so that people will focus their attention on information they might ignore otherwise. The existence of moral panic toward rave culture is supported by several studies in Canada [78], England [4], Ireland, [79], Australia [80], USA [81] and Israel [27]. Social agents, including the media, police and government, play a vital role in creating moral panic regarding the rave scene. The police persecution of Israeli rave parties over the years may have increased moral panic, resulting in stigmatization of rave culture [27].

The third theme refers to mental health, *negative experiences*, and relates to negative experiences following psychedelic drugs consumption at RMP. Participants described short-term reactions at RMP, including hallucinations and delusions. While qualitative sociological, ethnographic and anthropological studies have focused on subjective psycho-spiritual experiences at RMP which were perceived as positive for attendees, the current phenomenological inquiry indicates that subjective negative experiences are of notable concern among RMP attendees. This finding supports quantitative survey-based and case reports [34, 82], as well as a qualitative study [38], showing that attendees at RMP occasionally experienced negative drug-related reactions as a result of drug overdose or poly-substance abuse. Our finding is in accordance also with a quantitative [83] and a qualitative study [84] showing that in a recreational setting outside of RMP, whereby drugs are used with an intent of self-care, their consumption may also result for users in challenging experiences. This highlights the importance of the “setting” in psychedelic drug experience [85] and that without proper psychological support users may have subjective negative experiences. RMP are considered a particularly high-risk setting for potential drug-related challenging experiences due to factors including rave-specific environmental risks (i.e., humidity, hot temperature and mass crowd), lack of water (which could lead to dehydration), sleep deprivation and participants’ inexperience with psychedelic and other drugs [26, 38]. Few studies have shown that rave attendees who employed harm reduction strategies at RMP and used drug checking services, had lower prevalence of drug use at RMP and lower rates of drug-related adverse reactions [86, 87]. Consequently, employing harm reduction approaches and health-care services could provide support for individuals who have difficult experiences at the moments of the psychedelic effects and afterwards. Finally, integration of harm reduction programmes and health-care services may increase users’ positive attitudes toward these approaches, contribute to enhanced trust in policing, lower rates of drug use and lower odds of overdose and toxicity incidents [88].

The fourth theme*—positive experiences*—refers to participants’ positive experiences when combining rave music and psychedelics. These experiences could be divided into three categories: sensory, emotional and perceptual. The positive sensory effects included auditory, visual and tactile experiences. Participants described the influence of LSD and MDMA on their musical experience, including heightened auditory sensitivity, bodily reactions and the ability to see the words of songs penetrating their body. It may be that the experience of sensory enhancement is also due to the rave scene atmosphere which makes extensive use of photic stimulation of lasers, strobe lights and elaborate lighting systems, visual imagery projected onto screens and theatrical performances [23]. The ability to experience visual and tactile experiences during auditory stimulation when combining rave music with psychedelics, is in line with findings showing that auditory stimuli (pure sonic tones) combined with psychedelics induced a synesthesia-like effect (i.e., a condition in which a stimulus in one sensory modality consistently and automatically triggers concurrent percepts in another modality, see [89]), generating visual and tactile hallucinations that elevated participants’ sense of awe and beauty [90].

The positive emotional effects when combining rave music and psychedelics included positive feelings toward others, which were expressed through enhanced pro-social behaviour and empathy. This is in line with studies showing that RMP promotes feelings of love of self and others [14]. It is also in line with studies showing that engaging in a collective musical experience, especially when accompanied by movement, may result in enhanced social bonding [91], and that moving to a beat fosters pro-social behaviours such as trust, cooperation and empathy [92]. Pro-social behavior can be explained by Durkheim’s [93] sociological theory of *collective effervescence*. The theory contends that engaging in a shared ritualistic behaviour generates shared emotions, which can bring about a sense of union with others. The current finding are also consistent with studies demonstrating that even without the addition of music, MDMA [94] and LSD [95] may intensify pro-social behaviour and emotional empathy due to increase plasma oxytocin levels which may mediate the empathogenic effects of MDMA [96] and the emotional processing of LSD [9].

Another positive emotional effect found as a result of the rave music and drug combination was stress relief. It was expressed by the ability to vent difficult feelings, perceive rave parties as a time-out from everyday stress, and as relief from social constraints. These findings are in line with studies showing that young people attend rave parties to gain a sense of liberation and escapism from reality, obtain freedom from judgement and get away from their daily routine and every-day stress [97, 98]. Studies show that even without the addition of music, LSD [99] and MDMA [100] reduce emotional distress and alleviate the core symptoms of individuals coping with post-traumatic stress disorder, life-threatening illnesses, depression and anxiety. Thus, the addition of the musical component at rave scenes may have increased the relief effect, as meta-analyses of experimental studies show that listening to music while dancing reduces stress, anxiety and depression [101].

The positive perceptual experiences when combining rave music and psychedelics included self-acceptance, appreciation of life and connection to nature. Regarding self-acceptance, empirical evidence shows that the use of psychedelics such as LSD and MDMA, contribute to a more optimistic attitude toward the self [102]. The current findings showed that participating in a collective dance in nature may also increase self-acceptance, as it enhances self-image and subjective well-being. It has been shown that freestyle dance in the open-air fosters subjective well-being, including self-image and self-love [103].

Regarding appreciation of life, laboratory studies show that MDMA [104] and LSD [105] enhance positive mood, a positive attitude toward life and appreciation of life. This is also supported by a number of ethnographic studies showing that consuming psychedelics at RMP fostered a positive attitude toward life and higher appreciation of life [98, 106].

Finally, the current findings indicated connectedness to nature. This is in line with quantitative experimental, population-based and correlational studies [107, 108] that show association between psychedelic consumption and increased connectedness to nature. For example, Kettner et al. [108] found that lifetime consumption of psychedelic drugs was associated with a higher degree of self-identification and intimate connectedness toward nature. Regarding rave music, qualitative ethnographic [1] and anthropological [40] studies have found that RMP increased connectedness to nature. A possible explanation could be that being immersed in nature, in a free unrestricted environment, together with ecstatic dancing to the sounds of loud music, may facilitate the experience of an altered state of consciousness, thus contributing to greater appreciation of life, nature and the world in general [1, 14].

## Limitations and recommendations for future research

The present study had a number of limitations. First, participants were all native Hebrew speaking secular Jews (except for two bilingual English-Hebrew speakers and one observant Jew), mainly from the center of Israel. Israel is a diverse society, comprising various groups of Jewish religious identities, including secular, reform, conservative, orthodox and ultra-orthodox [109], as well as Muslim and Christian minorities. It would be of social and cultural value to expand this line of research and explore the subjective experience of rave party goers of different religious identities and of different Israeli minority groups such as Muslims and Christians. Second, all interviews were conducted via an on-line Zoom platform. While several studies have advocated Zoom interviews as an effective tool in building and maintaining rapport [56], some possible pitfalls have been pointed out. Low audio and visual-quality, and problems with internet connection for example, may contaminate the quality of the data [57]. Third, the study used retrospective accounts of participants’ experiences, and therefore, it is possible that some details of their experience may have been forgotten. It is highly recommended to conduct participatory research to gain a more profound understanding of participants’ lived experiences at RMP. Fourth, most of the participants in the current study were male. Since findings show gender differences in individuals’ responses to psychedelic drugs [110], future studies should ensure equal gender distribution of research participants to be able to address potential gender differences. Fifth, the current study did not explicitly enquire about the precise drug dosage used at RMP. Several studies suggest that factors related to “set and setting”, including drug dosage and poly-substance use, may influence drug-related challenging experiences [17, 26]. In the future, researchers should address this issue. Finally, the present study focused on rave music, a sub-genre of EDM. Future studies should look into other EDM scenes such as techno, house and rock.

## Conclusions and policy recommendations

The current study adds to scientific knowledge by highlighting the participants’ sense of insecurity due to Israel’s strict drug policy and the absence of harm reduction strategies at Israeli rave scenes. While many Western countries have adopted a drug-checking policy and have health management services on site at RMP, Israel has not embraced such policies. Harm reduction techniques that are used at RMP in Europe were shown to be effective in reducing and preventing consumption of impure drugs [111]. Therefore, it is recommended that Israeli policymakers consider a shift toward a more tolerant approach and adopt the European drug-checking policy to reduce negative consequences of drug use at RMP. Such a tolerant approach was expressed in a recent pilot programme that called for Israel’s Ministry of National Security, which oversees the Israel Police, to adopt a harm reduction policy and establish safe areas at authorized rave parties, despite the current illegal status of drugs in Israel [32].

Another novelty of the current study relates to the participants’ experience of stigmatization as drug addicts by society and law enforcement agencies simply by attending a rave scene. Reducing police presence and setting up intervention sites at RMP would not only make participants feel more at ease and free to enjoy the rave experience but could also potentially lessen the stigmatization of the scene and how it is perceived by the public. Additionally, a large drug campaign with information on drug use, drug content and dosage, consumption guidelines and possible adverse effects of specific drug combinations, could be a turning point in how rave parties are viewed by the general public and the authorities in Israel.

Adopting the European drug-checking and harm reduction policy at rave music festivals in Israel can assist in identifying drug “adulterants” and reduce overdose and death-related incidents. Finally, we hope that the findings of our study would expand the knowledge of policymakers on rave music and psychedelic drug consumption, which could be translated into safer and healthier experiences for the attendees at RMP.

## Data Availability

Data, materials and code will be available upon reasonable request. Raw verbatim data will not be available for ethical reasons.

## Footnotes

1 It is noteworthy, however, that Portugal currently holds an official drug decriminalization policy [63]. Decriminalization refers to a legislative process which removes prosecution against an action so that the action remains illegal but has no criminal penalties (for details, see https://www.law.cornell.edu/wex/decriminalization. Retrieved 2023, 15 November).
